# High dose-rate tandem and ovoid brachytherapy in cervical cancer: dosimetric predictors of adverse events

**DOI:** 10.1186/s13014-018-1074-2

**Published:** 2018-07-16

**Authors:** Kara D. Romano, Colin Hill, Daniel M. Trifiletti, M. Sean Peach, Bethany J. Horton, Neil Shah, Dylan Campbell, Bruce Libby, Timothy N. Showalter

**Affiliations:** 1 Department of Radiation Oncology, University of Virginia School of Medicine, 1240 Lee Street, Box 800383, Charlottesville, VA 22908 USA; 2 Division of Translational Research and Applied Statistics, Department of Public Health Sciences, University of Virginia School of Medicine, Charlottesville, VA USA

## Abstract

**Background:**

Brachytherapy (BT) is a vital component of the curative treatment of locally advanced cervical cancer. The American Brachytherapy Society has published guidelines for high dose rate (HDR) BT with recommended dose limits. However, recent reports suggest lower doses may be needed to avoid toxicity. The purpose of this study is to investigate incidence and predictive factors influencing gastrointestinal (GI) and genitourinary (GU) toxicity following HDR intracavitary brachytherapy for locally advanced cervical cancer.

**Methods:**

We retrospectively evaluated a cohort of patients with locally advanced cervical cancer who received CT-based HDR BT. Cumulative doses were calculated using the linear-quadratic model. Statistical analyses were used to investigate clinical and dosimetric predictors of GI and GU toxicity following HDR brachytherapy according to CTCAE v4.0 grading criteria.

**Results:**

Fifty-six women with FIGO IB1 – IVA cervical cancer were included. The overall rate of any GU adverse event (Grade 1+) was 23.3% (*n* = 13) and severe adverse events (Grade 3+) was 7.1% (*n* = 4). Of those, the bladder equivalent dose in 2- Gray (Gy) fractions (EQD_2_) D_2cc_ was ≥80 for three of the four patients. The overall rate of any GI adverse event was 26.8% (*n* = 15) and the rate of severe adverse events was 14.3% (*n* = 8). Of those, six of the eight patients had a rectal EQD_2_ D_2cc_ ≥ 65 Gy and seven patients had a sigmoid D2cc ≥ 65 Gy. Amongst clinically meaningful factors for development of adverse events (i.e. diabetes, smoking status, ovoid size, and treatment duration), there were no statistically significant prognostic factors identified.

**Conclusions:**

Severe adverse events are observed even with adherence to current ABS guidelines. In the era of recent multi-institutional study results, our data also supports more stringent dosimetric goals. We suggest cumulative D2cc dose limits of: less than 80 Gy for the bladder and less than 65 Gy for the rectum and sigmoid.

## Background

In the United States, cervical cancer continues to represent a sizeable portion of gynecological cancer burden among females, with an estimated 12,820 new cases in 2017 [1]. The definitive treatment for patients with locally advanced cervical cancer involves external beam radiotherapy (EBRT) and concurrent chemotherapy followed by a brachytherapy (BT) boost to achieve optimal treatment outcomes [2]. BT allows for dose escalation of the tumor in a conformal manner that minimizes the toxicity of nearby organs at-risk (OARs). This essential role of BT in the curative treatment paradigm has been confirmed by multiple reports, as it confers not only a local control but a survival advantage when compared to cohorts where EBRT is the only radiation treatment modality utilized [3, 4].

Over the past three decades, the use of high dose-rate (HDR) BT has substantially increased over low dose-rate (LDR) BT. HDR is now the predominant BT treatment modality in the United States [3, 5]. Our group recently reported equivalent local control and survival in our experience with LDR and HDR BT [6]. However, the incidence of severe toxicity temporarily increased shortly after the implementation of HDR BT at our institution [6]. The transient rise in severe toxicity represents a learning curve in the implementation of new technology and improvements were subsequently made including the incorporation of magnetic resonance imaging (MRI) to the image-guided BT (IGBT) workflow.

The American Brachytherapy Society (ABS) has published guidelines for the delivery of high quality image-guided HDR BT for locally advanced cervical cancer [7, 8]. Current guidelines recommend 3 dimensional (3D) IGBT with integration of ultra-sound, MRI, or computer assisted tomography to estimate dose to targets and OARs, and to ensure adequate tumor coverage. The total recommended 2 Gray (Gy) equivalent dose (EQD_2_) to target is 80–90 Gy combined dose from both EBRT and BT. There are variations in acceptable applicators (tandem and ovoid, cylinder, interstitial, etc.), dose specification, and dose fractionation, but dosimetry is required for each fraction due to the large fraction sizes and potential for toxicity. The ABS recommended OAR limits for bladder, rectum, and sigmoid are a D_2cc_ (dose to the hottest dose to 2 cm^3^ of tissue) of ≤90 Gy, ≤ 75 Gy, and ≤ 75 Gy, respectively [7, 8].

Similarly, the Group Europeen de Curietherapie (GEC) – European Society for Radiotherapy and Oncology (ESTRO) (GEC-ESTRO) gynecological (GYN) Working Group has published recommendations for the treatment of cervical cancer with IGBT [9]. Based on recent publications from the prospective multi-center, “European study on MRI-guided brachytherapy in locally advanced cervical cancer” (EMBRACE), study and retrospective “RetroEMBRACE,” the currently accruing EMBRACE II protocol OAR planning aims are D_2cc_ < 80 Gy for bladder, < 65 Gy for rectum, < 65 Gy for recto-vaginal point, and < 70 Gy for sigmoid and bowel [10, 11].

The purpose of this retrospective series is to report on our institution’s experience with image-guided HDR brachytherapy in the framework of the current guidelines set forth by the ABS and in light of the recent EMBRACE collaborative results supporting more stringent dose recommendations [7].

## Methods

### Patient population

After obtaining approval from the institutional review board, retrospective data was collected for patients with cervical cancer who received HDR tandem and ovoid BT and CT-based treatment planning from 2012 to 2014 to assess for dosimetric predictors of toxicity Clinical stages IB - IVA via the International Federation of Gynecology and Obstetrics (FIGO) staging criteria were included if dosimetry and follow up toxicity information was available during the specified time period. Patients were excluded if they received LDR BT or interstitial BT.

Baseline clinical prognostic factors were recorded including: age, stage (FIGO and tumor, nodal, metastasis (TMN) staging), Eastern Cooperative Oncology Group (ECOG) performance status, histology, clinical tumor size at diagnosis, diabetic history, and smoking status. Treatment-related variables including ovoid size and treatment duration were also chronicled. Clinical adverse events, if present, was scored by chart review as Grade 1–4 as per the common terminology criteria for adverse events (CTCAE) v4.03 for both acute and chronic conditions beginning from the start of radiation therapy [12]. Severe adverse events were defined as Grade 3 or higher.

### Treatment

#### External beam radiation therapy and chemotherapy

In 2012, our institution transitioned from LDR to a 3-D IGBT HDR program, and we have previously described our technique in detail [13]. In brief, the treatment of cervical cancer at our institution involves EBRT to a total dose of 45–50.4 Gray (Gy) in 1.8–2 Gy/fraction with concurrent cisplatin-based chemotherapy, if indicated, followed by intra-cavitary tandem & ovoid BT. BT is delivered after the EBRT course is complete, or in the last week of pelvic EBRT. The EBRT volume includes at least the whole pelvis with extended field technique included for more advanced disease such as positive pelvic or para-aortic nodes. Intensity modulated (IMRT) and 3-D techniques are used with 6–15 MV photon therapy. Positive nodes and/or involved parametria receive an additional boost dose of EBRT. We do not administer HDR BT on the same day as chemotherapy, and the goal is to complete all treatment within 56 days.

#### HDR brachytherapy

HDR BT is delivered with an ^192^Ir source and a single tandem and two symmetric ovoid applicators. The applicators are placed in a dedicated brachytherapy procedure suite with an in-room CT-on-rails system and HDR afterloader. A Smit sleeve is placed under anesthesia at the time of the first fraction of BT and remains in place through the final fraction. After placement of the applicators, all patients undergo computed tomography (CT) simulation for treatment planning. Beginning in 2014, our institution implemented a magnetic resonance imaging (MRI) integrated workflow for asynchronous MRI-based IGBT. Please see prior institutional publication for workflow details [13]. Pelvic MRI is obtained between the first and second fractions of BT in parallel and orthogonal planes of the Smit sleeve applicator. The MRI T2 weighted sequences define the extent of residual tumor and is co-registered along the plane of the Smit sleeve with the treatment planning CT for each subsequent fraction of BT. In accordance with ABS guidelines, the total HDR treatment dose is 25–30 Gy in 4–5 separate fractions (most commonly 5 fractions of 5–5.5 Gy each) with no more than two fractions per week and never on consecutive days. The goal for total tumor EQD_2_ is 80–90 Gy.

Manual dose optimization is performed on a standardized plan for individualized treatment planning. We start with a customary loading pattern for traditional Point A prescription, and then modify dwell positions and times to ensure coverage of the high-risk clinical target volume (HR-CTV) and the MRI-defined residual gross tumor volume (GTV-BT) and to minimize doses to the organs-at-risk (OAR). Since MRI is not available until after the first fraction of BT, an estimated HR-CTV is contoured on the planning CT even though an MRI is not available. Although HR-CTV is a concept based on MRI-based target delineation, contouring on CT for the first fraction permits us to track the cumulative doses in our clinical practice. To allow for treatment optimization based on daily OAR position and distension, critical OARs are contoured as a whole organ on the CT data set prior to treatment delivery for each fraction. A cumulative dose summation worksheet is used to track cumulative biologically effective doses (BED) and EQD_2_ from EBRT and each fraction of HDR BT for the targets and OARs. The alpha-beta ratio is assumed to be 10 for the tumor and 3 for normal tissues. Target doses include HR-CTV and GTV-BT V_100_, D_90_, and D_100_. OAR doses recorded include: bladder, rectum, sigmoid, and bowel D_0.1cc_, D _1cc_, and D_2cc_.

### Statistical analysis

Clinical, treatment-related, and toxicity factors were reported as categorical and continuous variables, as appropriate. Fisher’s exact test and the Wilcoxon Mann-Whitney test were used to assess differences in clinical and treatment characteristics between patient groups defined by those with adverse event grades of 2 or less and 3 or greater, with *p*-values presented in Table 1. Proportional hazard models and log-rank tests were used to identify clinical and dosimetric predictors, given in Table 1, for toxicity following HDR BT according to CTCAE criteria. All statistical analyses were performed using commercially available statistical software (SAS, version 9.4; SAS Institute Inc., Cary, NC).

**Table 1:** Baseline characteristics of all patients and by adverse event grade

	All	2 or less *n* = 49		3 or greater *n* = 7		
	n	n	% or 95% CI	n	% or 95% CI	*p*-value
Clinical characteristics						
Age at start of radiation treatment^1^	53.4	53.6	49.6–57.8	51.8	40.7–62.9	0.961
Histology		44	89.8	7	100	1.00
Squamous cell carcinoma	51					
Adenocarcinoma	4	4	8.2	0	0	
Adenosquamous	1	1	2.0	0	0	
FIGO stage		9	18.4	0	0	0.236
IB1	9					
IB2	14	13	26.5	1	14.3	
IIA1	0	0	0	0	0	
IIA2	1	1	2.0	0	0	
IIB	16	13	26.5	3	42.9	
IIIA	0	0	0	0	0	
IIIB	15	13	26.5	2	28.6	
IVA	1	0	0	1	14.3	
N stage		15	30.6	2	28.6	1.00
N0	17					
N1 – pelvic nodes	39	34	69.4	5	71.4	
M stage		39	79.6	7	100	0.719
M0	46					
M1 – para-aortic nodes	8	8	16.3	0		
Unknown	2	2	4.1	0		
Clinical tumor size at diagnosis (cm)^1^	5.4	5.4	4.6 – 6.1	5.5	4.3–6.7	0.742
ECOG		31	63.3	7	100	0.558
0	38					
1	9	9	18.4	0	0	
2	6	6	12.2	0	0	
3	2	2	4.1	0	0	
4	1	1	2.0	0	0	
Treatment characteristics						
Acute toxicity grade						–
0	45	45	91.8	0	0	
1	3	3	6.1	0	0	
2	1	1	2.0	0	0	
3	5	0	0	5	71.4	
4	2	0	0	2	28.6	
Chronic toxicity grade						0.008
0	36	35	71.4	1	14.3	
1	5	3	6.1	2	28.6	
2	5	4	8.2	1	14.3	
3	10	7	14.3	3	42.9	
4	0	0	0	0	0	
Treatment Response						0.914
Stable Disease	5	5	10.2	0	0	
Complete Response	20	18	36.7	2	28.6	
Partial Response	21	19	38.8	3	42.9	
Progression	5	4	8.2	1	14.3	
Unknown	4	3	6.1	1	14.3	
Treatment Duration						0.700
Greater than 56 days	27	23	46.9	4	57.1	
56 days or less	29	26	53.1	3	42.9	
Ovoid Size						0.236
Large	1	1	2.0	0	0	
Medium	15	15	30.6	0	0	
Mini	8	6	12.2	2	28.6	
Small	30	25	51.0	5	71.4	
Unknown	2	2	4.1	0	0	
Comorbidities						
Smoker		24	49.0	1	14.3	0.149
Never	25					
Former	9	8	16.3	1	14.3	
Current	22	17	34.7	5	71.4	
Diabetes Mellitus						1.00
No	50	43	87.8	7	100	
Yes	6	6	12.2	0	0	

*ECOG* Eastern Cooperative Oncology Group Performance Status, *FIGO* International Federation of Gynecology and Obstetrics

^1^Mean

## Results

### Clinical and dosimetric characteristics

Fifty-six (56) women with FIGO stage IB1 – IVA cervical cancer treated with HDR BT at our institution met inclusion criteria. Clinical and treatment characteristics are summarized in Table 1. The majority of patients in this cohort had FIGO stage IIB or IIIB (55.4%) squamous cell carcinoma and 39 patients (70.0%) had positive pelvic nodes noted on staging studies. Overall performance status was good; however, 3 patients were included with ECOG score of 3 or higher. The median follow up time, amongst patients alive at time of analysis, is 7 months (0–26 months).

The clinical and treatment characteristics of those without a severe acute adverse event (≤ Grade 2) or with a severe adverse event (≥ Grade 3) are presented in Table 1. Note that the estimated clinical tumor size at diagnosis for each group is similar (5.5 vs 5.4 cm). Patients with severe toxicity were more likely to be current smokers (71% vs 35%), have longer treatment duration (57% over 56 days versus 47%), more advanced T stage (all T2b or greater), and were slightly younger in age (52 vs. 54 years).

### Bladder toxicity

**Fig. 1:**
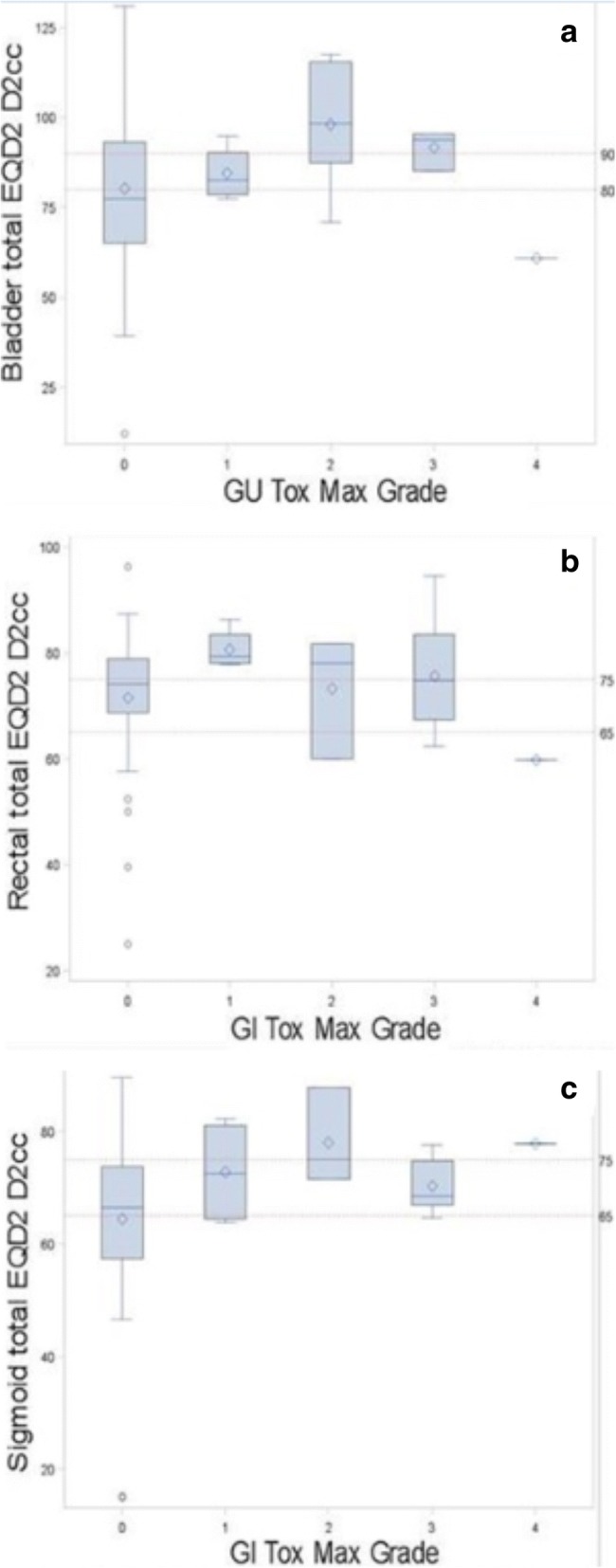
Box plots with adverse event and recommended dose constraints for bladder **a**, rectum (**b**) and sigmoid (**c**)

Overall, thirteen patients (23.3%) developed a genitourinary (GU) adverse event of any grade. Four patients (7.1%) experienced a severe (defined as Grade 3 or greater) GU adverse event. Among patients with Grade 3+ GU toxicity, 1 (25%) had bladder D2cc ≤80 Gy, 1 (25%) had bladder D2cc 80–90 Gy, and 2 (50%) >  90 Gy. A cumulative bladder D_2cc_ threshold of 90 Gy would permit a 5.6% rate of grade 3+ GU toxicity, compared to a 3.6% rate of grade 3 + GU toxicity if the threshold is lowered to 80 Gy. In Table 2, GU and gastrointestinal (GI) adverse events are reported by grade according to the EQD_2_ D_2cc_ for bladder, rectum, and sigmoid. Fig. 1 depicts the grade of adverse event by recommended dose constraints for bladder (A), rectum (B) and sigmoid (C).

**Table 2:** Frequency of adverse event by recommended dose category

	Bladder total EQD2 D_2cc_						All	
	0 to 80		80 to 90		> 90			
	n	%	n	%	n	%	n	%
GU Toxicity Grade	24	85.7	5	62.5	14	70.0	43	76.8
0								
1	2	7.1	1	12.5	1	5.0	4	7.1
2	1	3.6	1	12.5	3	15.0	5	8.9
3	0	0.0	1	12.5	2	10.0	3	5.4
4	1	3.6	0	0.0	0	0.0	1	1.8
All	28	100.0	8	100.0	20	100.0	56	100.0
	Rectal total EQD2 D_2cc_						All	
	0 to 65		65 to 75		> 75			
	n	%	n	%	n	%	n	%
GI Toxicity Grade	8	72.7	17	85.0	16	64.0	41	73.2
0								
1	0	0.0	0	0.0	4	16.0	4	7.1
2	1	9.1	0	0.0	2	8.0	3	5.4
3	1	9.1	3	15.0	3	12.0	7	12.5
4	1	9.1	0	0.0	0	0.0	1	1.8
All	11	100.0	20	100.0	25	100.0	56	100.0
	Sigmoid total EQD2 D_2cc_						All	
	0 to 65		65 to 75		> 75			
	n	%	n	%	n	%	n	%
GI Toxicity Grade	20	90.9	11	57.9	10	66.7	41	73.2
0								
1	1	4.5	1	5.3	2	13.3	4	7.1
2	0	0.0	2	10.5	1	6.7	3	5.4
3	1	4.5	5	26.3	1	6.7	7	12.5
4	0	0.0	0	0.0	1	6.7	1	1.8
All	22	100.0	19	100.0	15	100.0	56	100.0

*GU* Genitorinary, *GI* Gastrointestinal, *EQD2* Equivalent dose in 2 Gy fractions assuming α/β = 3 Gy, *D*_*2cc*_ the most irradiated 2 cm^3^ of normal tissue volume

### Rectal/sigmoid toxicity

Overall, fifteen patients (26.8%) developed a GI adverse event of any grade. Eight patients (14.3%) developed a severe GI adverse event. Of the 8 patients with Grade 3+ GI toxicity, 2 had rectal D_2cc_ of 65 Gy or less (25%), 3 had doses between 65 and 75 Gy (37.5%), and 3 had doses greater than 75 Gy (37.5%). A cumulative rectal D_2cc_ threshold of 75 Gy would result in a 16.1% rate of Grade 3+ GI toxicity. A cumulative sigmoid D_2cc_ threshold of 75 Gy would permit a 14.6% rate of grade 3+ GI toxicity compared to a permitted rate of 4.5% if the threshold is lowered to 65 Gy.

### Prognostic factors for toxicity

Logistic regression models were used to model GU and GI adverse events. The models were adjusted by dose to related organs (bladder for GU toxicity and rectum/sigmoid for GI toxicity) in additional 5 Gy increments. No covariates were statistically significant (*p* = 0.276 for bladder, *p* = 0.361 for rectum, and *p* = 0.092 for sigmoid). Several other factors of interest were assessed for their prognostic effects on time to developing Grade 3+ adverse events, including diabetes, smoking status, ovoid size, and treatment duration. Log-rank testing did not show any of the curves to approach statistical significance (*p*-values > 0.05).

### Dosimetry

**Fig. 2:**
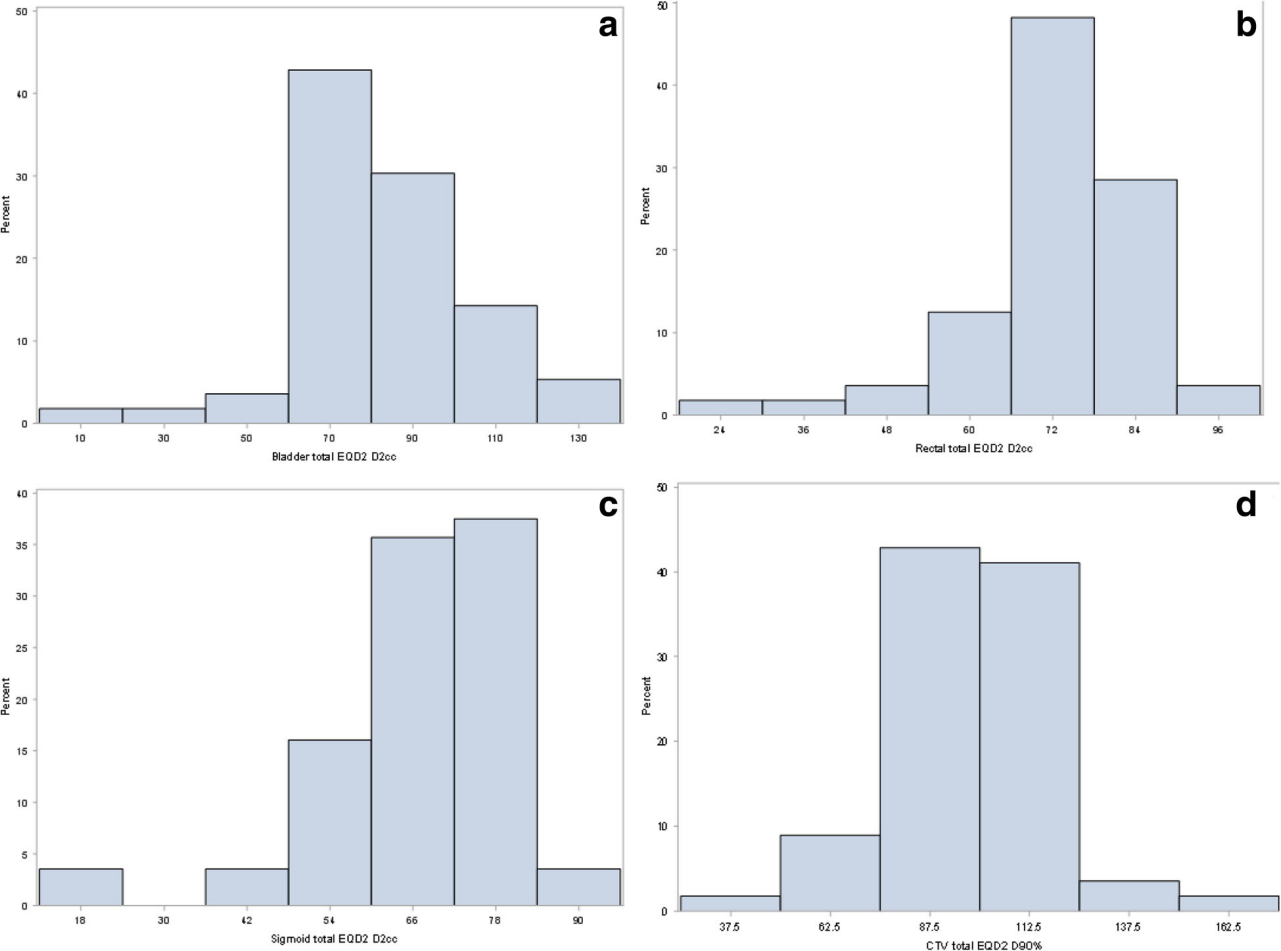
Histograms percent of patients with equivalent dose in 2 Gy fractions (EQD2Gy) for bladder **a**, rectum **b**, sigmoid **c**, and CTV (**d**)

Figure 2 depicts in a histogram the percentage of patients receiving specified EQD2 doses.

## Discussion

The ABS guidelines in 2012 recommended EQD2 D_2cc_ dosimetric thresholds of 90 Gy for the bladder and 75 Gy for the rectum and the sigmoid to minimize toxicity [7]. Our data demonstrates that high rates of toxicity are still possible in clinical practice even with adherence to the current ABS guidelines for dose limits to normal tissues. Based on our observed toxicities, we suggest more stringent cumulative D2cc dosimetric goals than in current ABS guidelines: specifically, less than 80 Gy for the bladder and less than 65 Gy (or as low as achievable without compromising local control) for the rectum and sigmoid.

Our study is relevant in light of other recent multi-institutional publications also supporting more stringent dose limits. The prospective multi-center EMBRACE study was initiated in 2008 utilizing MRI-guided BT prior to an understanding of specific OAR dose constraints in this 3-D era. Subsequent reports from EMBRACE and the retrospective RetroEMRACE studies report an overall severe toxicity (Grade 3 of higher) of around 3–7% per organ [10, 11]. A dose-volume effect for rectum was established with D_2cc_ ≤ 65 Gy correlating with more minor and less frequent rectal morbidity where rectal D_2cc_ ≥ 75 Gy was associated with more severe rectal morbidity and risk of fistula [14]. Significant dose effect curves for bladder morbidity have also been reported with a 5% risk of late urinary morbidity for D_2cc_ 60–70 Gy [15]. Based on these findings, the currently accruing EMBRACE II protocol OAR planning aims are D_2cc_ < 80 Gy for bladder, < 65 Gy for rectum, < 65 Gy for recto-vaginal point, and < 70 Gy for sigmoid and bowel [10].

There are inherent challenges to comparing toxicity outcomes across different series. The American Brachytherapy Task Group conducted a meta-analysis of retrospective and prospective series from 2000 to 2015 [16]. In their report, the late GI and GU toxicities ranged from 4 to 11% and 1–6%, respectively, for radiation alone and 1–11% and 2–20%, respectively, for chemo-radiation. The authors note that only a limited number of image-based brachytherapy (IGBT) series reported toxicity. Further, there is a the lack of consistency in toxicity scoring systems and other confounding variables such as differences in treatment planning, which may lead to significant variation in dosimetric outcomes to the surrounding OAR. Our rate of severe GI adverse events is higher than those summarized in this meta-analysis; which may be related to the above-mentioned limitations with differences in scoring, reporting, and the inclusion of patients in the pre-MRI based imaging era.

### Rectal/sigmoid toxicity

We found a high rate of severe GI toxicity for rectal and sigmoid D_2cc_ over 65–75 Gy. Other series in the era of IGBT treatment planning have validated volume-based dose parameters EQD2 D_2cc_ as a reliable parameter for predicting rectal and bladder toxicities [15]. Increased rates of rectal toxicity (acute, late, and/or both) have been demonstrated with a D_2cc_ of 78 Gy and of 65 Gy [15, 17, 18]. Another retrospective series of patients identified a significant relationship between late rectal toxicity and a cumulative rectal dose in EQD2 greater than 65 Gy. Taking into account these various reports in the literature and our rectal toxicity rate, it is reasonable to suggest that a more stringent cumulative D_2cc_ threshold would lead to more optimal toxicity outcomes.

The dose constraints for the sigmoid are still unclear. There was no sigmoid 2-D correlate and clinical data for sigmoid are not as widely reported. While there has not been a sigmoid dose-volume relationship established in the previously published literature, this cohort demonstrates an association between sigmoid doses > 65 Gy and high rates of severe toxicity. Thus, current protocols including EMBRACE II indicate respecting the same OAR dose constraint for rectum and sigmoid [19].

### Bladder toxicity

A well-defined dose-effect relationship for bladder doses and urinary morbidity in the treatment of cervical cancer has been demonstrated. D_2cc_ thresholds of 100 Gy and 95 Gy have been shown to correlate with bladder toxicity in previously reported series [15, 20]. However, another series of IGBT reporting a 5.9% rate of late grade 3+ GU morbidity but could not identity a relationship between GU toxicity and the bladder D_2cc_ [17]. An additional point to consider with bladder OAR is the highly distensible nature of the organ and that dose-volume histogram (DVH)-summed parameters may not always accurately estimate bladder dose [21]. Review of the literature clearly suggests that the limit of the bladder dose threshold has yet to be clearly defined, but our results suggest considering the fact that a D_2cc_ dose of 80 Gy will permit more acceptable toxicity rates than the current dosimetric guidelines for the bladder. The EMBRACE II protocol defines the bladder contour as the outer wall including the bladder neck with a planning aim of D_2cc_ < 80 Gy with a hard limit of < 90 Gy [19].

### Vaginal toxicity

Until recently, late gynecologic toxicity was rarely reported. In a recent meta-analysis, only 1 of 16 prospective trials reported late gynecologic toxicity with a mean rate of 16% following RT alone. From EMRACE we learned that recto-vaginal point dose correlated with vaginal stenosis with 65 Gy leading to 20% vaginal stenosis [22]. While vaginal toxicity is not reported here, these data again support tighter dose constraints to limit toxicity.

### Image guidance

The incorporation of IGBT for applicator placement and treatment planning has generated the opportunity for much advancements in target coverage and normal tissue sparing. Ideal dosimetric distributions with HDR can be achieved only if an optimal optimization process accompanies proper applicator placement. With the evolution of treatment planning processes from prescribing a dose to a fixed point to 3-D treatment planning, the development of individualized dosimetric distributions that adequately cover the HR-CTV while sparing the OAR can be challenging. However, multiple studies have demonstrated the benefit of 3-D treatment planning with improvements in local control and survival endpoints *and* better morbidity outcomes [16, 23, 24]. Due to its demonstrated benefits, a growing number of institutions have made the switch to IGBT in recent years, but it has yet to be universally implemented due to the complexity and time-consuming nature of IGBT [16]. For institutions that have the capability to perform hybrid CT-MRI or MRI-based IGBT, the superior target-volume delineation that is attainable with MRI [9, 25] has the potential to allow for compliance to more stringent OAR dose thresholds, potentially allowing for better morbidity.

### Limitations

The limitations of this study are inherent to the retrospective nature of a single institution’s experience. Moreover, we did not control for variations in applicator placement accuracy, target volume size, or differences in systemic therapy. We attempted to identify clinical factors prognostic for whether patients would develop adverse events but were unable to do so due to the relatively small sample size of patients in our cohort experiencing grade > 3 adverse events. As mentioned, scoring systems for toxicity are inconsistent across series and other series may report their data using different toxicity scales.

We were able to identify a clinically meaningful relationship between dosimetric limits for normal tissues and toxicity, but perhaps due to small sample sizes we were unable to also identify clinical factors that would be significantly prognostic of whether patients would experience severe adverse events. For example, additional factors that may influence toxicity in this patient cohort that were not reported here include obesity, co-morbidity index (heart disease, COPD, hypertension, etc.), and baseline GU/GU functional repots. Future studies can incorporate these potentially important factors.

### Future directions

The currently accruing EMBRACE II study is evaluating image guided adaptive BT in a prospective multi-institutional setting [19]. The results of this trial will help us to further understand the dose and volume effect relationships of OARs and related morbidity. One of the ways this trial will improve upon prior experiences is through the implementation of a wider variety of applicator types (intracavitary and interstitial), which will allow for greater modulating ability of source position and dwell times. Additionally, organ manipulation (i.e bladder filling or rectal retraction) can confer a gainful benefit to achieving better dosimetric distributions [26, 27]. Continued development of applicators to displace normal tissues has the potential to further improve normal tissue doses and related morbidity. Despite the importance of toxicity and the need to prioritize optimization of dosimetry to spare organs-at-risk, it is also important to remember that delivering sufficient dose to the cervical tumor must remain the top priority in the curative treatment of cervical cancer. In some situations, therefore, threshold doses to adjacent organs may be exceeded in order to achieve adequate tumore dose.

## Conclusion

In clinical practice, a relatively high rate of GI and GU toxicity is still possible even with adherence to current ABS guidelines for dosimetric objectives. We suggest considering more stringent cumulative D2cc dosimetric goals than in current ABS guidelines: less than 80 Gy for the bladder and less than 65 Gy (or as low as achievable without compromising local control) for the rectum and sigmoid.

## Abbreviations

3D: 3-dimensional
ABS: American Brachytherapy Society
BED: Biologically effective dose
BT: Brachytherapy
CTCAE: Common terminology criteria for adverse events
D_0.1cc_, D_1cc_, D_2cc_: Dose delivered to the hottest 0.1 cc, 1 cc, and 2 cc of an organ at-risk
D_90_, D_100_: Dose delivered to the hottest 90 and 100% of the target volume
EBRT: External beam radiation therapy
ECOG: Eastern Cooperative Oncology Group
EMBRACE: European study on MRI-guided brachytherapy in locally advanced cervical cancer
EQD_2_: 2 Gray equivalent dose
FIGO: International Federation of Gynecology and Obstetrics
GEC-ESTRO: Group Europeen de Curietherapie (GEC) – European Society for Radiotherapy and Oncology (ESTRO)
GTV-BT: MRI-defined gross tumor volume at time of brachytherapy
Gy: Gray
GYN: Gynecological
HDR: High dose-rate
HR-CTV: High-risk clinical target volume
IGBT: Image-guided brachytherapy
IMRT: Intensity-modulated radiation therapy
LDR: Low dose-rate
MRI: Magnetic resonance imaging
OAR: Organ at-risk
V_100_: Volume of target volume receiving 100% of the prescription dose
